# Nd:YAG 1064-nm laser for residual infantile hemangioma after propranolol treatment

**DOI:** 10.1038/s41598-023-33870-0

**Published:** 2023-05-08

**Authors:** Z. Khamaysi, N. Pam, H. Zaaroura, E. Avitan-Hersh

**Affiliations:** 1 Department of Dermatology, Rambam Health Care Campus, Haifa, Israel; 2 Bruce Rappaport Faculty of Medicine, Technion Institute of Technology, Haifa, Israel

**Keywords:** Medical research, Energy science and technology

## Abstract

Infantile hemangiomas (IH) are common benign tumors of infancy. Most IH involute, either spontaneously, or secondary to pharmacological treatment with systemic propranolol. Propranolol treatment mostly leads to regression of hemangiomas with satisfactory aesthetic results, but unfortunately not in all cases. To assess the safety and efficacy of long pulsed Nd:YAG 1064 nm laser in treating patients with residual infantile hemangioma after systemic propranolol treatment. This is an open-label prospective cohort study. 30 patients with focal residual IH that had sub-optimal responses to systemic propranolol treatment were enrolled in the study. The patients were treated with 1 to 3 sessions with long pulsed Nd:YAG 1064 nm laser. The maximal response of the IH was assessed using a 4-point scale evaluation scale system. Of the 30 patients enrolled, 18 patients exhibited a great response (> 76% improvement), 10 patients had a good response (> 51–75% improvement), while only 2 patients showed a moderate response (< 50% improvement) to the treatment. No patients had an unsatisfactory response. No serious side effects were observed, and only minor side effects were reported. The treatment with long pulsed Nd:YAG 1064 nm laser for residual IH, which were resistant to systemic propranolol treatment, is safe and effective. Thus, we suggest its use as a second-line treatment for patients with sub-optimal aesthetic results following systemic propranolol.

## Introduction

Infantile hemangiomas (IHs) are the most common infantile soft-tissue tumors. The incidence of IHs is unknown, with estimates of occurrence varying from 3 to 10% of the population^1–4^. Multiple studies had shown that not all IHs completely resolve spontaneously. Without intervention, approximately 15–40% of IHs will involute with resultant textural skin changes^5–8^.

Since 2008, the use of propranolol revolutionized the treatment of IHs and became the standard of care, with high efficacy rates and minimal side effects. However, many IHs involute only partially, even after systemic treatment of propranolol. The exact cause of partial resolution of IHs after systemic propranolol treatment has not been established, although some theories have been suggested^9^.

Residual hemangioma often leads to cosmetic disfigurement and dissatisfaction. The patients’ legal guardians often seek additional individualized treatments such as laser or surgery to improve the cosmetic result of the residual IH. Laser treatment for residual IHs has been routinely practiced since 1990 with various laser modalities demonstrating diverse results. Laser operation by Nd:YAG was first demonstrated by J.E. Geusic et al. in 1964^10^. Since the early 2000s, long pulsed (LP) Nd:YAG laser, within the millisecond time range, has been considered a particularly safe and effective treatment for various vascular lesions. The Nd:YAG LP laser causes photocoagulation of vascular lesions including IHs, dilated blood vessels, prominent facial capillaries and spider hemangiomas^11–13^. The Nd:YAG laser's active medium is a crystal of yttrium, aluminum, and garnet doped with neodymium ions. By functioning in the near-infrared region of the electromagnetic spectrum at a wavelength of 1064 nm, hemoglobin and oxyhemoglobin act as the absorbing chromophores, while the vessel walls are the target by means of heat diffusion from the inner vessel towards its walls. In addition, water in the vessel walls acts as an additional chromophore to absorb the laser. Hemoglobin has an absorption coefficient curve that peaks at 418 nm, 524 nm, 577 nm, and again at 1064 nm^14^.

One of the most limiting factors in laser treatment of vascular lesions is the lesion depth from the skin surface. Currently, a wavelength of 1064 nm can reach a maximum of 5-10 mm. Other wavelengths commonly used, such as 590–595 nm pulsed dye lasers (PDL), being shorter in wavelength, can reach a maximum depth of 1.2 mm. These shorter wavelengths are successfully able to target superficial lesions such as Port-wine stains and superficial hemangiomas. For deeper vascular lesions like deep hemangiomas a wavelength of 1064 nm is commonly used^15,16^. Additionally, 1064 nm infrared light is more readily absorbed by oxyhemoglobin with low absorption by melanin, triggering photothermolysis of the hemoglobin chromophore while avoiding damage to the epidermis.

The therapeutic result of LP 1064 nm laser treatment for residual IH is dependent upon the response of the individual residual hemangioma. The results vary from patient to patient, and depend upon the size and type of the residual hemangioma lesion.

## Patient and methods

### Study population (Fig. 1)

**Figure 1:**
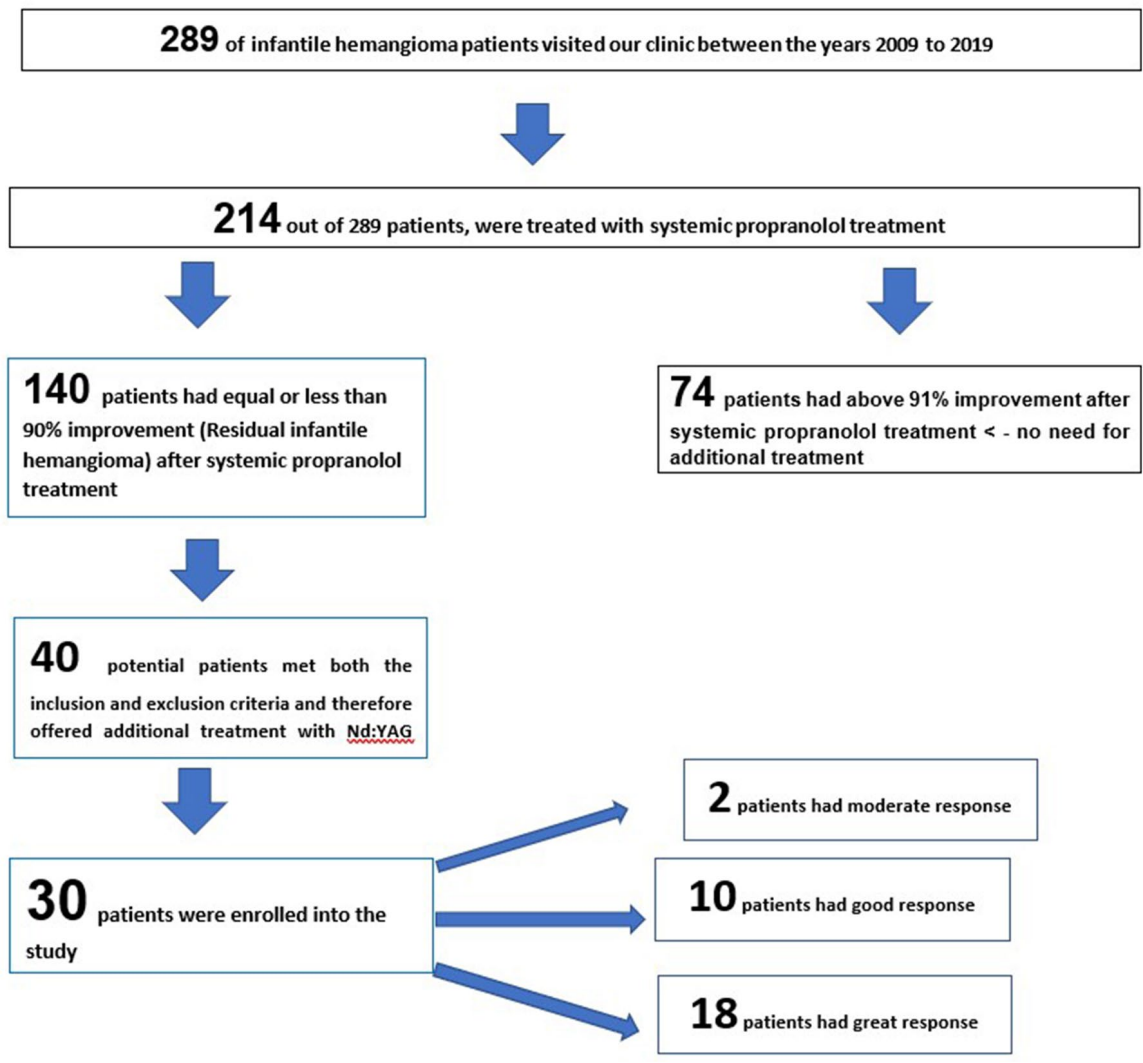
Study design and response rate.

Between January 2009 and December 2019, 289 patients with a diagnosis of IH visited our outpatient clinic. Of them, 214 (74%) patients were treated with systemic propranolol. Treatment with systemic propranolol was indicated due to; existing ulcerations, risk of bleeding, potential loss of function of an essential organ or cosmetic disfigurement. The response was evaluated using a 4-point visual scale^17^. 74 (35%) patients out of 214 patients had a final 4-point visual score above 90% and did not require any additional treatment. 140 (65%) patients had a final 4-point visual score of 90% or less at the end of the systemic propranolol treatment and did not achieve maximal satisfactory aesthetic results. Out of those 140 patients, 30 (21.42%) agreed to be enrolled in our study and received additional treatment with Nd:YAG LP 1064 nm laser.

### Laser therapy methods

Before, during and post laser treatment, we performed external cooling with the Cryo 6, a cold-air chiller device (Zimmer, Neu-Ulm, Germany), which decreases the skin temperature quickly and results in a lower risk of skin burns whilst allowing a constant parameters for the entirety of the treatment. Cooling with Cryo 6 was performed intermittently and not continuously, 10 min before, 10 min during and 10 min after the laser treatment, with no additional local or general anesthesia. We used Nd:YAG LP 1064 nm laser (Harmony XL PRO, Alma Laser, Nürnberg, Germany). All laser sessions were with an energy density of 150–450 J/cm^2^, a pulse duration of 10–45 ms, and spot size within a range of 2–6 mm, which was determined according to the size and depth of the residual hemangioma. The patient’s eyes were covered with block-out glasses with adjustable nosepieces. The physician wore OD + 6 laser protective goggles. The end point for color changes was grey color that evolves in the hemangioma.

The laser therapy protocol included one to three laser sessions per patient. The decision to have one or more laser sessions was made by the principal investigator according to the patient response and level of improvement. If the patients’ did not achieve a satisfactory result, ‘great’ according to the 4-point evaluation scale, after a single laser session, they were offered additional sessions. Time intervals between sessions were 2 months for all patients.

All legal guardians of the patients’ were informed about potential side effects such as swelling, erythema, blistering and skin erosion. The patients’ parents gave written informed consent including photo documentation for use in the study, education, research and publication purposes. The protocol was approved by the ethics committee of the Rambam healthcare campus (0512-19-RMB). All methods were performed in accordance with the relevant guidelines and regulations.

### Assessment of clinical response

Photographic documentation was performed both before and after each laser session and was assessed by a single dermatologist (the principal investigator).

The final response rate results were evaluated one month following the last laser session according to the 4-point visual scale. This scale was proven to be a reliable tool to evaluate response to treatment for residual IHs, according to various studies^17^, and included: change in the size of the residual lesion, color, degree of vascularization, texture, atrophy, topographical abnormalities, pigmentary changes and overall cosmetic appearance. The assessment was categorized into four groups:

1. Great response = 76–100% regression of residual IH compared to initial residual lesion
2. Good response = 51–75% regression of residual IH compared to initial residual lesion
3. Moderate response = 26–50% regression of residual IH compared to initial residual lesion
4. Poor/unsatisfactory response = 0–25% regression of residual IH compared to initial residual lesion

Side effects were evaluated immediately after every sessions and 6 months following the last session.

#### Patients' satisfaction survey

6 months after the completion of the final laser treatment, the parents completed a survey regarding their satisfaction from the laser treatments as follows: Very unsatisfied, unsatisfied, neutral, satisfied and very satisfied.

### Data analysis

The data was analyzed using SPSS software version 25. Statistics were performed using means and standard deviations (SD) for the continuous variables, and frequencies and rates for the discrete variables. Differences were assessed using Kruskal–Wallis and Mann–Whitney tests for the independent continuous variables and chi-square tests for the discrete variables. Wilcoxon Sum Rank tests were conducted for assessing the differences between dependent variables. Spearman tests were conducted for assessing correlations. A multivariate model was conducted using linear regression. Statistical significance was considered for *p* values < 0.05.

### Ethics approval

The protocol was approved by the ethics committee of the Rambam healthcare campus (0512-19-RMB).

## Results

### Patient characteristics (Table 1)

**Table 1:** Baseline characteristics of patients and their infantile hemangioma (IH).

	Number	Percentage
Gender		
Male	10	33.3
Female	20	66.7
(IH) type		
Superficial	21	70
Deep	9	30
IH distribution		
Face	10	33.3
Neck	2	6.66
Limb	6	20
Trunk	11	36.6
Genitals	1	3.33

In total, 30 patients were enrolled in our study, 20 female (66.7%) and 10 male (33.3%). The average age at the beginning of the first laser sessions was 24.6 months (± 11.52). The majority of the residual, focal IH were superficial and located on the face and trunk.

#### Response rate

**Figure 2:**
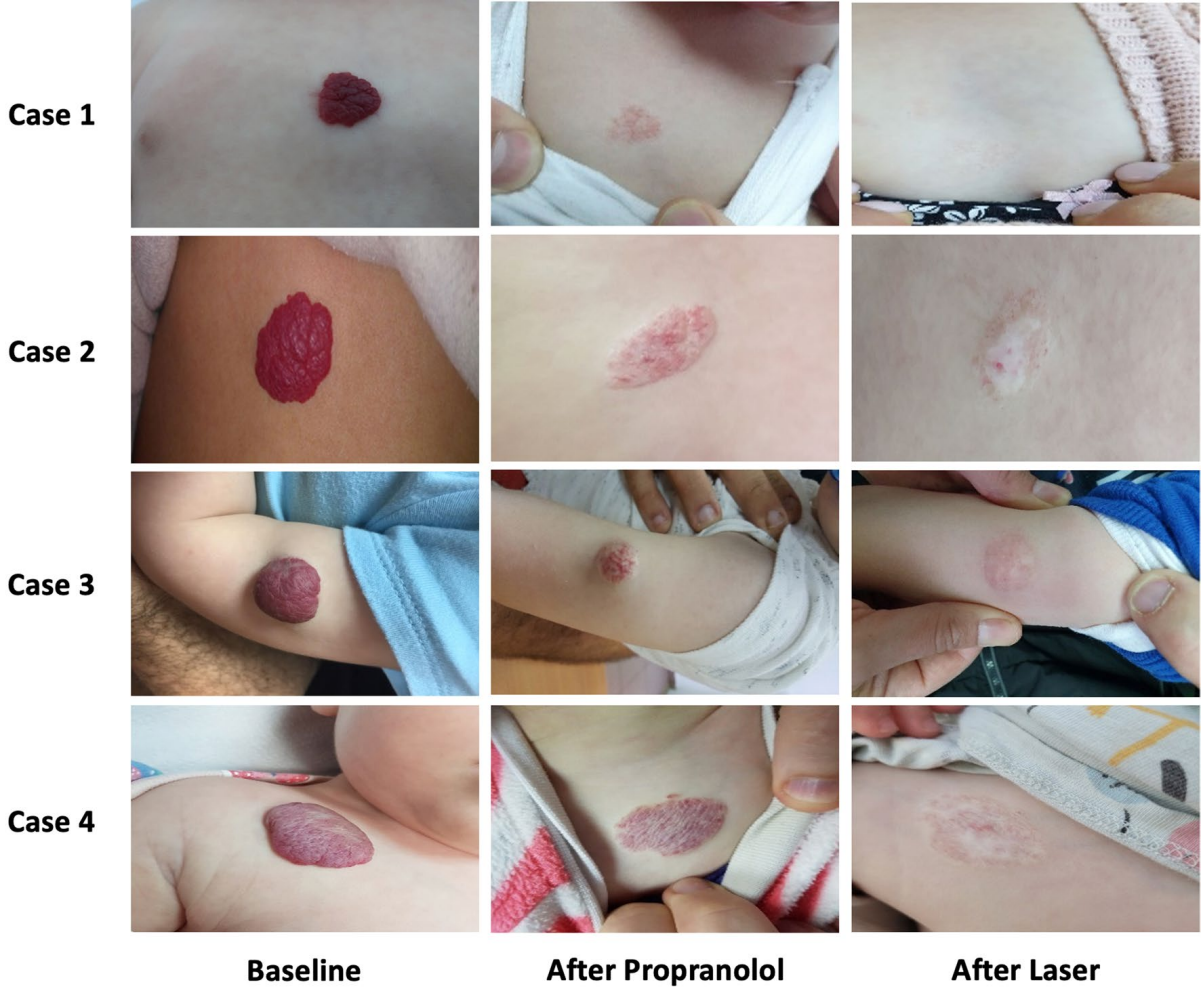
Patient's response to laser after propranolol treatment.

6 patients (20%) received a single laser session, 10 patients (33.3%) received two sessions and 14 patients (46.7%) received three sessions. 76% of patients had a pulse width of 10 ms (ranges 10–45). The average fluence was 172.00 J/cm2 (ranges 150–450). Eighteen (60%) patients exhibited a great response (Fig. 2), 10 (33.3%) patients achieved a good response, 2 (6.6%) patients had a moderate response. None of the patients had a poor response to the treatment. In total, 28 patients (93.3%) achieved a combined final 4-point visual scale result of moderate response or above.

### Patients' satisfaction survey from overall laser treatment (Fig. 3)

**Figure 3:**
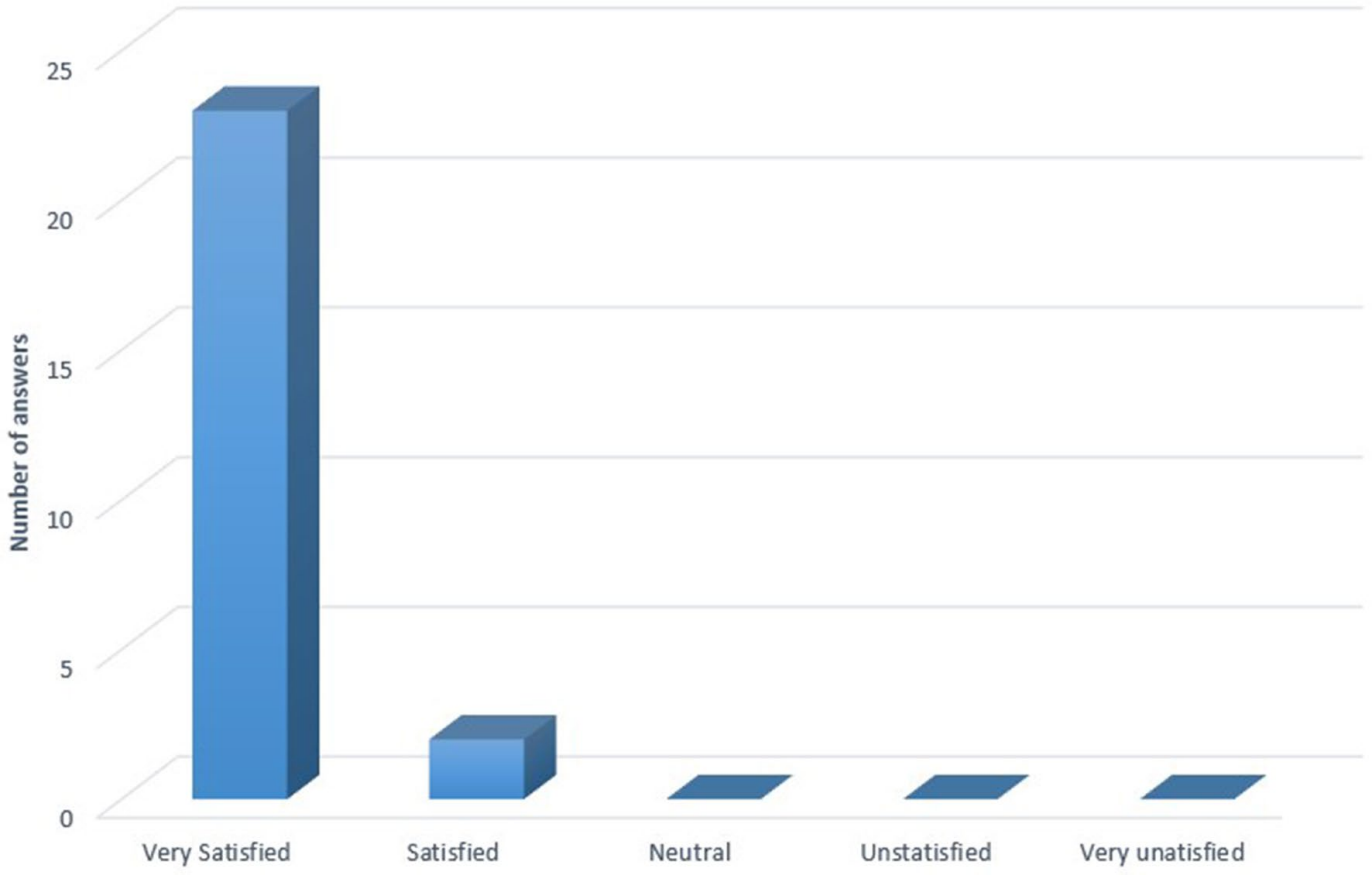
Patients' satisfaction survey from overall laser treatment.

In total, 25 (83.3%) out of 30 parents of patients enrolled in the study participated in our survey. 23 parents (92%) reported being ‘very satisfied’ and the remaining two parents (8%) said they were "satisfied" with the treatment outcome.

### Factors affecting the response rate

**Figure 4:**
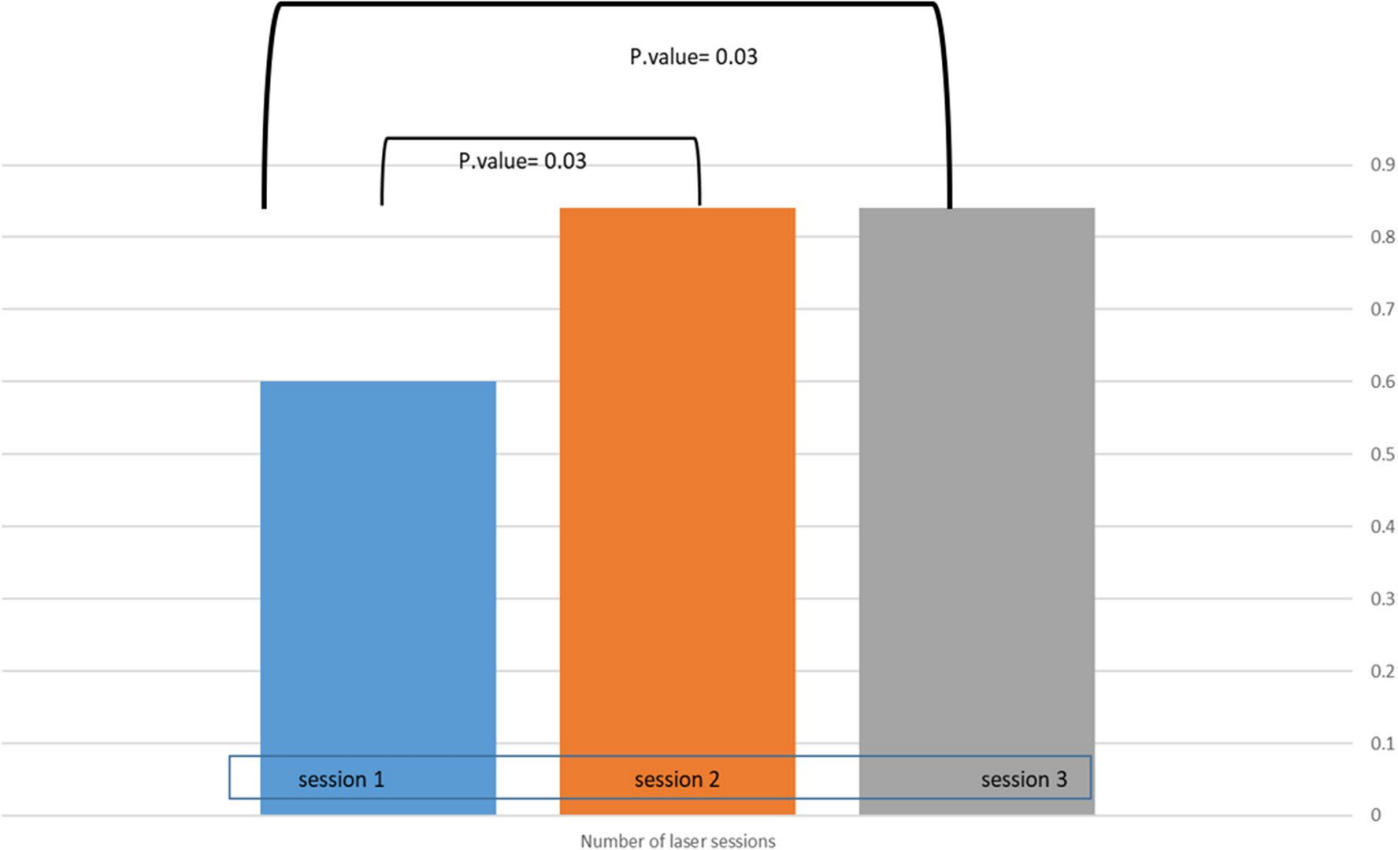
Response rate according to the number of laser sessions.

While searching for prognostic factors that may affect the response rate to Nd:YAG LP laser treatment, we found a significantly better outcome in patients receiving more than one laser treatment (Fig. 4). Patients who had a single treatment (0.63 ± 0.20) had significantly lower response rates in comparison with patients who had two or three laser treatments (0.87 ± 0.17), (*p* = 0.03). However, there was no significant difference between two and three treatments.

In addition, we found that a larger spot size was associated with better response rates. Specifically, patients treated with spot size of 6 mm had higher Visual scale (0.90 ± 0.13) in comparison with patients treated with a spot size 2 mm (0.85 ± 0.17), (*p* = 0.04).

Other factors such as the depth of residual IHs, age of initial systemic propranolol treatment and age of first laser treatment did not affect the overall response rates (Table2).

**Table 2:** Summary of correlations for predicting response rate for Nd:YAG 1064-nm laser.

	B	Std error	β	t	*p*
Gender	− 0.19	0.11	− .45	− 1.69	.11
Deep lesion	0.07	0.12	.18	0.63	.53
Age at the beginning of the first laser session	0.01	0.01	.36	0.86	.41
Maximal fluence	0.00	0.00	.26	0.76	.46
Ulcerated lesion	− 0.01	0.26	− .01	− 0.03	.98
Age at the beginning of systemic propranolol treatment	0.01	0.01	.21	0.86	.40
Response rate at the end of systemic propranolol	1.10	0.63	.56	1.74	.10

In addition, we found that the localization of the lesion did not affect response (Table 3).

**Table 3:** Association between the anatomical location of infantile hemangioma and response rate outcome for Nd:YAG 1064-nm laser treatment.

	No	Yes	*p* value
	M ± SD	M ± SD	
Face	0.81 ± 0.21	0.83 ± 0.17	0.85
Neck	0.82 ± 0.20	0.85 ± 0.07	0.97
Limb	0.77 ± 0.20	0.92 ± 0.13	0.08
Trunk	0.82 ± 0.17	0.80 ± 0.22	0.88
Genitals	0.82 ± 0.19	0.7	0.62

### Safety and side effects

Side effects are presented in Table 4. All patients had erythema and edema that resolved spontaneously within 3 days from each treatment and endured minimal pain during the sessions, which resolved immediately post treatment. 14 of the patients had loose skin (46.7%) and 8 had a residual hypopigmented scar (26.7%). Less common side effects were residual hyperpigmentation (N = 2, 6.7%) and blisters (one patient, 3.3%). None of the patients suffered from infection or nerve injury.

**Table 4:** Incidences of side effects after treatment (out of 30 patients).

Side effect	Frequency (patients)	Rate
Erythema and edema	30	100%
Minimal pain	30	100%
Residual hypopigmented scar	8	26.7%
Residual Hyperpigmentation	2	6.7%
Loose skin	14	46.7%
blister	1	3.3%
Infection	0	0
Nerve injury	0	0

## Discussion

Infantile hemangiomas (IH) are common benign tumors of infancy, most of them resolve during early infancy, some persist and require treatment with systemic propranolol oral solution, which mostly leads to significant improvement. However, in our recent studies we note that some IHs remain with unsatisfactory results^18,19^.

In addition, after initial successful propranolol treatment, recurrent IHs occur in up to 25% of treated children. In 10–15% of these patients, repeated treatment with propranolol was required^20–30^. Furthermore, in rare instances, propranolol can cause side effects such as hypoglycemia, hypotension, bradycardia, diarrhea and hyperkalemia. Therefore, in such cases, there is an unmet need for additional management following the propranolol treatment.

The laser therapy option is especially important in providing additional treatment modalities for residual and recurrent hemangiomas as well as for patients with contraindications or at risk of side effects to propranolol. Numerous studies on PDL treatments have shown response rates of 80–90%^13,14^. Given that PDL is characterized by selective absorption by hemoglobin, it can achieve effective photothermolysis of vascular lesions. Yet, the maximum penetration depth of PDL is 0.75–1.2 mm^31^. 40–50% of IHs extend into the subcutis, emphasizing the need for a deeply penetrating laser, such as Nd:YAG laser (1064 nm, penetration depth of 5–6 mm) to effectively treat IHs^31,32^. Additionally, PDL can transform oxyhemoglobin into methemoglobin and lead to thrombus formation^32^. The absorption of Nd:YAG laser by methemoglobin and thrombi is a further advantage of this laser system. This can enable effective vessel destruction while sparing the surrounding tissues^32^.

Two large studies on the combination therapy with PDL and ND:YAG laser for the treatment of IH have been published to date. Saafan and Salah treated 25 patients with IH in the head and neck region and reported that 72% healed completely, 16% healed with mild hyperpigmentation/loss of pigmentation, or “structural skin changes,” and 12% responded inadequately^32^. In a retrospective study by Alcántara-González and colleagues, all 22 patients benefited from PDL and Nd:YAG LP laser treatment, irrespective of the stage of IH development^31^.

To the best of our knowledge, this is the first published prospective data that studies the treatment of residual hemangioma with Nd:YAG LP laser monotherapy. Out of 30 patients that were enrolled in our study, 28 patients had a great or good response. Along with the physician assessment of treatment success, our study also surveyed the parents asking about their satisfaction. These results suggest high efficacy with a low rate of side effects and are superior to those reported in PDL monotherapy studies^31^.

In our study, all patients had minimal, spontaneously resolving side effects after 3 days from the last laser sessions. 6 months following the last sessions, about one third of the patients had loose skin, a quarter had a residual hypopigmented scar and minor cases of residual hyperpigmentation or blister. In other published studies, laser therapy was associated with few side effects; only a small number of patients developed mild atrophy, ulcerations or hyperpigmentation^31,32^.

Various cooling systems have been developed to protect the epidermis against thermal damage^33^. Application of ice or chilled water may also be used to cool the blood vessels in the upper dermis, however limiting the effects of the laser. Our laser therapy was performed with Zimmer Cryo 6 cold-air chiller device. Cryo 6 decreases the skin temperature quicker and maintains a constant dosage throughout the entire treatment, protecting the epidermis from skin burns. Moreover, it could replace the need for anesthesia, considering many local anesthetics formulas contain sympathomimetic and vasoconstrictors, which may diminish the effectiveness of laser treatment.

Of importance, it was found that one treatment was not enough, a better outcome was reported in patients who had two sessions. However, the third treatment did not give an additional advantage. This suggests that for the majority of patients’ two laser sessions of Nd:YAG LP laser will be sufficient for significant improvement, in comparison to PDL monotherapy for IHs which usually requires more than 5 sessions^13^. Thus, treatment with Nd:YAG LP laser reduces the number of necessary treatments, while ensuring an adequate response.

Regarding the factors affecting the response rate, efficacy did not depend upon gender, age, or the depth of the lesion, but was affected by the laser spot size and number of sessions.

The lack of difference in response rates between deep and superficial IHs may be explained by choosing a deeply penetrating laser wavelength (Nd:YAG laser 1064 nm). Laser of larger spot size was associated with better response. Larger spots size has deeper penetration, which is in line with our hypothesis that more better responses to treatment are due to the depth of laser penetration, ie. Nd:YAG laser compared to other laser systems.

In summary, Nd:YAG LP laser therapy (1064 nm), has been shown to be an effective and safe method for the treatment of IHs, with only 1–2 sessions needed. Our results suggest that Nd:YAG LP laser may be recommended as second-line therapy for residual IHs after propranolol treatment, especially for IHs with a deep component.

## Data Availability

The datasets used and/or analysed during the current study available from the corresponding author on reasonable request**.**
